# Effect of refining time on the physicochemical and functional properties of palm-sunflower based anhydrous cream produced in a stirred ball mill

**DOI:** 10.1016/j.fochx.2025.102972

**Published:** 2025-08-29

**Authors:** Mohammad Salama, Birsen Sarıcı, Avni Çakıcı

**Affiliations:** a Department of Food technology, Istanbul Aydın University, P.O. Box 65, 00014 Istanbul, Turkey; b Department of Health Sciences, Duzce University, Düzce, Turkey; c Department of Toxicology, İstanbul Aydın University, P.O. Box 65, 00014 Istanbul, Turkey

**Keywords:** Anhydrous fat cream, Palm oil, Particle size distribution, Viscosity, Stirred ball mill

## Abstract

This study investigated the effect of refining time on the physicochemical and functional properties of anhydrous cream prepared from a palm-sunflower oil blend using a stirred ball mill. Refining was performed for 0–300 min, and its impact on particle size distribution, rheology, oxidative stability, and thermal behavior was assessed. The target particle fineness (D90 ≤ 30 μm) was achieved at approximately 180 min, with negligible reduction thereafter. Refining progressively increased apparent viscosity and decreased the flow behavior index, confirming stronger shear-thinning behavior. Oxidative stability and melting transitions remained stable across treatments, demonstrating that lipid integrity was preserved under controlled processing conditions (45 °C). An optimal refining duration of 180–240 min provided the desired fineness and viscosity without excessive energy consumption. These findings define cost-efficient, scalable processing parameters for producing additive-free, trans-fat-free cream systems with reliable rheological, oxidative, and thermal stability, supporting industrial applications in confectionery, spreads, and frozen desserts.

## Highlights

• Particle fineness (D90 ≤ 30 μm) achieved after 180 min of stirred ball mill refining.
• Rheological analysis confirmed enhanced consistency and pseudoplastic behavior.
• Oxidative markers remained stable across refining times, indicating lipid integrity.
• Thermal profiles were unaffected by refining duration, confirming melt stability.
• Optimal refining duration supports clean-label, trans-fat-free cream formulation for industrial applications.

## 1 Introduction

Anhydrous cream systems are critical for texture, flavor release, and shelf stability in premium confectionery and frozen desserts. Their structure finely dispersed solid particles such as sugars, milk powders, or cocoa suspended in a continuous lipid phase, allows modulation of spreadability, mouthfeel, and oxidative resistance (Biglarian et al., 2022; Wang et al., 2023). Functional performance is governed by fat phase composition and processing conditions, particularly solid fat content (SFC) and fat droplet coalescence, which dictate rheology, sensory attributes, and stability (Zhen et al., 2025; Zhou et al., 2022). Intermediate SFC values, in particular, promote partial coalescence during oral processing, enhancing smoothness and mouth coating (Shewan et al., 2021; Wang et al., 2023).

In response to global restrictions on partially hydrogenated oils (PHOs), demand has increased for natural, trans-fat-free fat systems with reliable functional properties (Zhen et al., 2025). Palm oil (PO), characterized by a balanced solid fat content (SFC), high oxidative stability, and a favorable β' polymorphic form, provides desirable plasticity and spreadability, making it a technically viable substitute for PHOs (Sulaiman et al., 2022). When blended with sunflower oil (SFO), PO can yield structured fat systems with optimized melting behavior and improved textural attributes (Malvano et al., 2024). Moreover, the use of stirred ball mill refining allows precise control of particle size distribution while improving energy efficiency (Martelli & Di Nunzio, 2025).

Refining in stirred ball mills is a pivotal step for fat-based dispersions, reducing particle size below the sensory threshold (∼25 μm) to improve texture and uniformity (Chantanuson et al., 2022; Santosh et al., 2023). While widely studied in chocolate and dairy emulsions (Elbendari & Ibrahim, 2025; Turk-Gul et al., 2023), its impact on trans-fat-free, vegetable oil-based anhydrous creams remains poorly understood. Excessive refining may also cause over-shearing, elevated temperatures, and altered fat crystallization, compromising both oxidative stability and overall processability (Ghanaatpishehsanaei & Pal, 2023; Mohammadi et al., 2024). Thus, optimizing refining time is essential to balance technological efficiency with nutritional and sensory quality.

To address this knowledge gap, the present study systematically evaluates how refining duration (0–300 min) in a stirred ball mill affects particle size distribution, rheological behavior, oxidative stability, and thermal properties of a palm oil-sunflower oil cream. We hypothesize those variations in refining time influence particle dispersion and rheological interactions, thereby modulating cream consistency, melting behavior, and oxidative resistance. By defining the processing window that balances technological efficiency with both product quality and nutritional value, this work provides new insights to guide the development of clean-label, trans-fat-free cream systems tailored for confectionery, spreads, and frozen dessert applications.

## 2 Materials and methods

This study was initially conducted within a previously implemented academic program at the Food Processing Laboratory of Istanbul Aydın University. The experiments were subsequently reviewed and adapted under doctoral research supervision, with specific analyses performed at the Food Engineering Laboratories of Yıldız Technical University. Refining was conducted using a laboratory-scale stirred ball mill at the Central Research Laboratories of Yıldız Technical University (Türkiye), operated under standardized confectionery conditions to maintain consistent shear and thermal exposure. Each 10 kg batch of cream was processed in a batch mode for durations ranging from 0 to 300 min. The temperature and shear rate were continuously monitored and, when necessary, adjusted through the machine's digital interface in accordance with the experimental design.

**Fig. 1:**
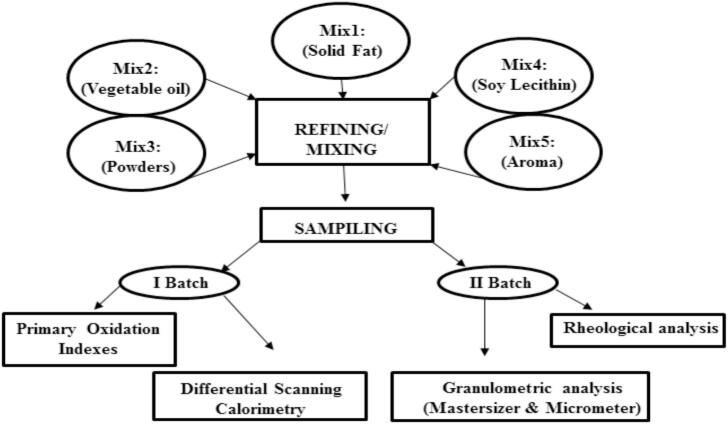
Mixing steps of cream preparation.

The ingredients used for anhydrous cream production were provided by Sandra Ice Cream Company (Hebron, Palestine) and shipped to Türkiye for use in the experiments. A total of five ingredient mixes, prepared in sequential mixing steps, were combined to produce a 10 kg batch, as illustrated in Fig. 1. All ingredients were commercially sourced and food-grade. In Mix 1, cocoa butter and palm oil were blended to form the solid fat matrix. Mix 2 contained sunflower oil, additional vegetable oils, and hazelnut cream, contributing to product spreadability and oxidative stability. Mix 3 comprised the dry phase, sugar, glucose syrup, skim milk powder, whole milk powder, lactose, powdered cream, maltodextrin, and milk proteins, to enhance body and texture. Mix 4 incorporated soy lecithin as an emulsifier to promote solid dispersion in the lipid phase and to provide mild antioxidant protection. Lastly, Mix 5 included flavoring agents to enhance aroma and taste. After refining, the cream was stored in containers at 25 °C before further analysis. Table 1 presents the list of ingredients, allergens, and the physicochemical and nutritional information of the finished product.

**Table 1:** Ingredients and nutritional value of the final product.

Chemical / physical characteristics	Value	Unit	Nutritional information	100 g of product	Unit
Humidity	1.5	%	Energy	∼531.56	Kcal
Fats	39		Fat	39	g
Ash	1.5		Saturated fat	8.3	
			Protein	0.14	
			Carbohydrates	45	
			Sugars	46	
			Salt	–	

### 2.1 Characterization of anhydrous cream

The prepared creams were analyzed to determine their final physicochemical and nutritional properties after refining, and additional characterization was conducted on samples collected at predefined refining intervals (0, 5, 10, 15, 20, 30, 40, 60, 70, 90, 120, 150, 180, 210, 240, 270, and 300 min). Sample quantities were minimized to minimize interference with the refining process.

#### 2.1.1 Particle size measurement

Two complementary techniques were employed to assess particle size during the refining process. The first method used an electronic digital micrometer (Model: Mitutoyo MDC-25SX, measurement range: 0–25 mm, accuracy: 0.001 mm), a widely used approach for rapid, high-precision assessment of larger particle dimensions in fat-based systems such as chocolate and confectionery creams (Rad et al., 2019). The instrument was calibrated according to the manufacturer's guidelines before use. A small quantity of cream sample was placed on the fixed anvil, and the spindle was gently advanced until initial mechanical resistance was detected, after which the particle size was read directly from the digital display. Measurements were conducted in two modes: in-line during refining at 45 °C and offline after conditioning samples at 25 °C for 24 h. For each sample, five replicate measurements were taken to ensure measurement repeatability. While this approach provides immediate readings of individual particle dimensions, it does not provide comprehensive information on the overall particle size distribution.

To obtain a complete particle size profile, a Mastersizer laser diffraction particle size analyzer (Malvern Hydro 2000MU, Malvern Instruments Ltd., UK) was employed using a wet dispersion methodology optimized for anhydrous food suspensions (Mureşan, 2018). This technique, based on light scattering and laser diffraction principles, enables precise and reproducible measurement of particle sizes across a range of 0.01–3500 μm. Approximately 0.1 g of each cream sample, collected at different refining times, was dispersed in sunflower seed oil (refractive index = 1.496) at room temperature (20 ± 2 °C). For each sample, three independent replicates were analyzed, and each replicate comprised 20 consecutive measurements. Granulometric analysis was performed using the instrument's dedicated software, and results were reported as D10, D50, and D90 values, representing the particle diameters below which 10 %, 50 %, and 90 % of the sample volume, respectively, are contained.

#### 2.1.2 Rheometer analysis

The viscosity of the cream samples was determined using a rotational rheometer (Anton Paar Rheolab QC, Austria) equipped with a standard vane geometry for semi-solid food systems and operated under controlled shear conditions. Measurements were performed over a shear rate range (γ•) of 0.01–100 s^−1^, a range commonly applied to characterize the flow and textural behavior of fat-based food products. The resulting flow curves were fitted to the Power Law model to obtain the consistency index (K) and the flow behavior index (n), which describes the pseudoplasticity and viscosity response of the creams. The rheological behavior was modeled using the Power Law equation, expressed as follows:

(3) ὴ=K∙γ˙^n−1

where ὴ is the viscosity (Pa·s), K is the consistency index (Pa·s^n^), γ• is the shear rate (1/s) and n is the dimensionless flow behavior index*.*

#### 2.1.3 Primary oxidation indices

To assess the initial oxidative stability of the anhydrous cream formulated with palm oil, the primary oxidation indicators, namely free fatty acid (FFA) content and peroxide value (PV), were determined. Cream samples were collected at refining times of 0, 90, 150, and 300 min. At each time point, 10 g of cream were transferred into Falcon tubes and centrifuged using a Beckman Coulter Allegra X-30R refrigerated centrifuge at 10,000 rpm for 20 min to separate the lipid phase. The recovered supernatant fat was then subjected to a second centrifugation at 14,000 rpm for 10 min to remove residual solids. All centrifugation steps were carried out at ambient temperature, and analyses were conducted on freshly separated oil.

The free fatty acid (FFA) content was determined by titration with a sodium hydroxide (NaOH) solution in ethanol/ether, with phenolphthalein as the indicator, and expressed as the percentage of oleic acid using the following formula:

(1) $$\%\mathrm{acidity}\;\left(\mathrm{in oleic acid}\right)=\left(V*N*P/10*m\right)$$

where, V = is the volume of sodium hydroxide (mL).

N = is the normality of the sodium hydroxide solution.

P = is the oleic acid equivalent weight = 282.

m = the weight, in grams, of the sample used for the analysis.

The peroxide value (PV), expressed in milliequivalents of active oxygen per kilogram of oil, was determined by the standard iodometric titration method, in which hydroperoxides oxidize potassium iodide to iodine, followed by titration with sodium thiosulfate using starch as the indicator. The PV was calculated at the end of the titration according to the following eq. (2):

(2) $$\mathrm{Number of peroxides}\;\left(\frac{\mathrm{meqO}2}{\mathrm{kg}}\mathrm{oil}\right)=\frac{\left(V\;x\;N\;x\;1000\right)}{m}$$

V = is the number of mL of the sodium thiosulphate solution used for the test.

N = is the normality of the sodium thiosulphate solution used for titration.

m = the weight in g of the substance to be analyzed.

FFA and PV were selected as internationally standardized and sensitive indicators of early lipid degradation in fat-based systems. FFA quantifies hydrolytic rancidity through liberated fatty acids, while PV quantifies lipid hydroperoxides, the primary intermediates of oxidative rancidity (Zhang et al., 2021). Both parameters have been extensively validated across vegetable oils, oleogels, and structured fat matrices (Geng et al., 2023; Shuai et al., 2024). Sampling intervals (0, 90, 150, and 300 min) were designed to capture both unrefined and progressively refined states, thereby enabling detection of both oxidation onset and its progression, consistent with prior refining studies on fat dispersions (Elbendari & Ibrahim, 2025; Turk-Gul et al., 2023). Analytical conditions, including sample mass (m), titrant normality (N), and endpoint volume (V), followed AOCS methods (Cd 8b-90 for PV; Ca 5a-40 for FFA) and ISO protocols (3960:2017; 660:2020), which have undergone interlaboratory validation, ensuring robust and comparable results across different lipid matrices.

#### 2.1.4 Differential scanning calorimetry

A differential scanning calorimeter (DSC Q20, TA Instruments, USA) was used to evaluate thermal transitions and melting behavior of the cream samples. Samples collected after 0 and 300 min of refining were analyzed to assess thermal stability and their suitability for ice cream applications. Approximately 8 mg of each sample was hermetically sealed in aluminum pans and loaded into the DSC cell. The analysis consisted of two sequential thermal cycles: (i) an isothermal hold at 60 °C for 1 min followed by cooling at 10 °C min^−1^ to ˗80 °C; and (ii) an isothermal hold at ˗80 °C for 30 min followed by heating at 10 °C min^−1^ to 60 °C. Thermograms were processed using Universal Analysis software (TA Instruments) to determine onset, peak, and endset melting temperatures, together with the melting enthalpy (ΔH, J/g fat). These parameters were used to define the melting profile and to evaluate the thermal robustness of the cream matrix for potential ice cream applications.

### 2.2 Statistical analysis

In order to assess model fit quality, the regression coefficient (*R*^*2*^) was calculated. One-way analysis of variance (ANOVA) followed by Duncan's multiple range test was applied to determine whether differences among sample means were statistically significant (*p* ≤ 0.05). The residuals were analyzed to verify the assumption of normality. Data analysis was performed using SPSS Statistics for Windows, version 17.0 (SPSS Inc., Chicago, IL, USA).

## 3 Results and discussion

### 3.1 Granulometric analysis

**Fig. 2:**
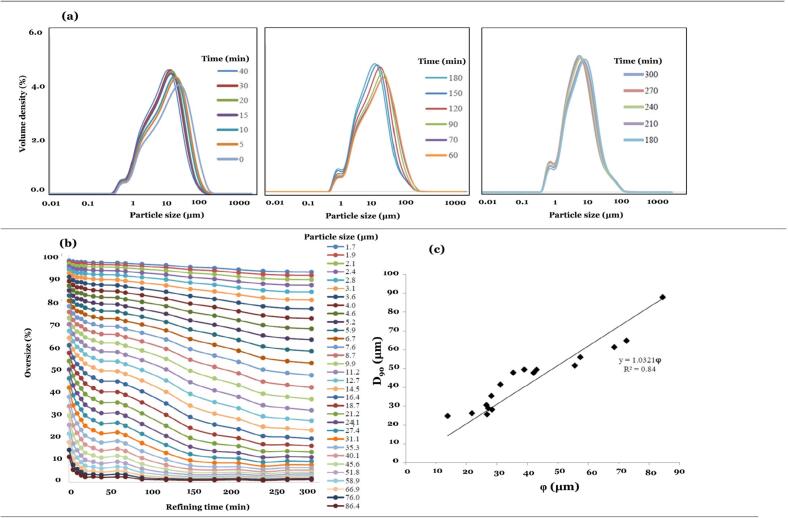
(a) particle size distribution of cream stored at 25 °C after 0, 5, 10, 15, 20, 30, 40, 60, 70, 90, 120, 150, 180, 210, 240, 270, 300 min of refining, (b) Cumulative oversize fraction (%) as a function of refining time for different particle size classes (1.7–864.4 μm); each curve corresponds to a class midpoint, and values represent the proportion of particles larger than the indicated size at each refining point, and (c) the correlation between D_90_ values vs fineness (ϕ).

The granulometric distribution of anhydrous cream strongly influences its structural integrity, texture, and sensory quality; therefore, particle size is a critical parameter in refining process optimization. In this study, particle size distribution (PSD) was monitored at refining intervals ranging from 0 to 300 min using laser diffraction analysis (Fig. 2a). Initially, the PSD curve exhibited a unimodal profile with volume-weighted mean diameters above the fineness threshold, indicating a coarse but homogeneous dispersion. As refining progressed, the PSD curve gradually shifted leftward, reaching an average particle diameter of ∼20 μm at 180 min. Beyond this point, further size reduction was minimal, and the PSD tended to stabilize, suggesting that particle fragmentation under the fixed 4 kW energy input of the stirred ball mill had approached its mechanistic limit, beyond which additional stress produced negligible effects.

These observations are consistent with the findings of Elbendari and Ibrahim (2025), who likewise observed unimodal PSD stabilization in stirred ball mill applications. Similarly, Bolenz et al. (2014) and Bolenz and Manske (2013) reported that ball mill-refined chocolates exhibit a stable unimodal distribution with negligible improvement beyond prolonged refining. The diminishing returns in size reduction were further evidenced by Svanberg et al. (2011), who showed that solid particle incorporation and pre-crystallization in chocolate model systems altered microstructural dynamics, necessitating careful time-energy optimization. Collectively, the present results align with these reports, indicating that refining durations beyond 180 min, while technically possible, provide limited technological advantage and incur unnecessary energy costs.

The cumulative frequency distributions in Fig. 2b provide further insight into particle-size evolution during refining. Particles ≤10 μm showed only modest reduction in the first 60 min, followed by a rapid decline between 60 and 240 min, indicating slower fragmentation kinetics for finer fractions. In contrast, coarse particles (>30 μm) decreased sharply within the first 40 min, after which further reduction was negligible. The intermediate fraction (10–30 μm) exhibited a steady linear decline throughout the entire process. These patterns confirm a size-dependent fragmentation mechanism, whereby coarse particles disintegrate rapidly under early high-shear conditions, while finer particles require extended mechanical input to achieve significant reduction.

Statistical analysis of PSD parameters confirmed these trends. Table 2 summarizes the evolution of D10, D50, and D90 values during refining. D90 values obtained by Mastersizer remained nearly unchanged after 120 min, confirming the asymptotic trend of size reduction. Complementary micrometer measurements taken at 45 °C (online) and 25 °C (after 24 h) offered additional validation, with slightly higher D90 values recorded at 25 °C. This increase is likely due to partial fat crystallization, which alters refractive index and surface morphology, thereby increasing apparent particle size. Despite these variations, a strong correlation between micrometer and laser diffraction data was observed (*R*^*2*^ = 0.84; Fig. 2c), demonstrating good methodological agreement.

**Table 2:** Master sizer parameters (D_10_, D_50_, D_90_) and fineness of creams digital micrometer during 300 min of refining time.

	Digital micrometer		Master sizer		
Refining time (min)	D_90_ (45 °C)	D_90_ (25 °C)	D_90_	D_10_	D_50_
0	80.40 ± 2.71^g^	84.40 ± 3.37^i^	87.80 ± 0.60^l^	3.55 ± 0.03^k^	22.93 ± 0.16^o^
5	66.00 ± 1.58^f^	72.60 ± 2.42^h^	64.70 ± 2.11^k^	3.26 ± 0.02^j^	18.36 ± 0.47^n^
10	62.80 ± 9.16^f^	68.60 ± 1.56^h^	61.30 ± 1.33^j^	3.17 ± 0.06^ij^	17.53 ± 0.37^m^
15	44.40 ± 2.54^e^	57.40 ± 1.40^g^	55.96 ± 0.58^hi^	3.17 ± 0.02^ij^	16.50 ± 0.11^l^
20	38.80 ± 2.67^de^	55.60 ± 1.63^g^	51.53 ± 0.28^gh^	3.09 ± 0.27^hi^	15.46 ± 0.03^k^
30	31.80 ± 1.35^cd^	43.00 ± 1.14^f^	49.33 ± 0.69^gh^	3.16 ± 0.16^gh^	14.63 ± 0.08^j^
40	29.20 ± 1.24^bc^	42.00 ± 0.94^f^	47.60 ± 1.36^g^	3.00 ± 0.03^gh^	14.10 ± 0.23^i^
60	23.60 ± 0.40^abc^	39.00 ± 0.89^ef^	49.36 ± 0.21^g^	2.96 ± 0.02^fg^	13.96 ± 0.12^hi^
70	23.40 ± 1.77^abc^	35.40 ± 0.40^de^	47.70 ± 0.28^f^	2.90 ± 0.05^f^	13.46 ± 0.06 ^h^
90	23.80 ± 1.39^abc^	31.20 ± 0.49^cd^	41.43 ± 0.12^e^	2.79 ± 0.02^e^	12.50 ± 0.10^g^
120	20.40 ± 1.43^ab^	28.20 ± 0.63^c^	35.40 ± 0.26^d^	2.63 ± 0.01^d^	11.10 ± 0.00^f^
150	24.40 ± 1.72^abc^	26.60 ± 1.47^c^	30.56 ± 0.08^d^	2.46 ± 0.01^cd^	9.63 ± 0.01^e^
180	20.60 ± 1.32^ab^	27.20 ± 0.66^c^	28.83 ± 0.14^cd^	2.34 ± 0.01^bc^	8.85 ± 0.01^d^
210	18.80 ± 1.06^a^	28.40 ± 2.01^c^	28.13 ± 0.42^bc^	2.20 ± 0.03^b^	8.23 ± 0.09^c^
240	20.00 ± 1.14^a^	26.80 ± 1.59^c^	25.60 ± 0.05^a^	2.09 ± 0.08^ab^	7.55 ± 0.08^b^
270	20.80 ± 1.77^ab^	21.80 ± 0.80^b^	26.23 ± 0.54^ab^	2.04 ± 0.02^a^	7.29 ± 0.08^ab^
300	17.80 ± 0.73^a^	13.80 ± 0.86^a^	24.76 ± 0.37^a^	2.01 ± 0.07^a^	6.96 ± 0.11^a^

Values in the same column followed by different letters differ significantly at *P* < 0.05 level (Duncan's method).

These findings are consistent with previous studies. Fidaleo et al. (2017) observed significant differences in D90 values of anhydrous chocolate creams milled for 195 and 225 min, with reductions from 37.6 to 35.2 μm and corresponding fineness values near 23 μm. In the present study, comparable D90 stabilization was achieved after 200 min, indicating that refining had reached a functional threshold. Similarly, Wong et al. (2024) noted that while refining decreases D90 values, the reduction rate slows markedly beyond a critical duration, largely attributed to fat film formation on particle surfaces and limited fragmentation capacity. This so-called “plateau effect” was also evident in the current system, where although a slight further decrease in D90 was recorded at 300 min, the marginal improvement offered negligible benefits for product homogeneity while imposing disproportionate energy demands, underscoring the importance of optimizing refining time.

Overall, the granulometric data highlight that an optimal refining duration of approximately 180 min is sufficient to achieve the desired PSD and fineness in anhydrous cream, rendering further milling technologically unnecessary and economically inefficient. This represents a critical optimization that ensures desirable texture and sensory quality while enhancing production efficiency in industrial applications.

### 3.2 Rheological analysis

**Fig. 3:**
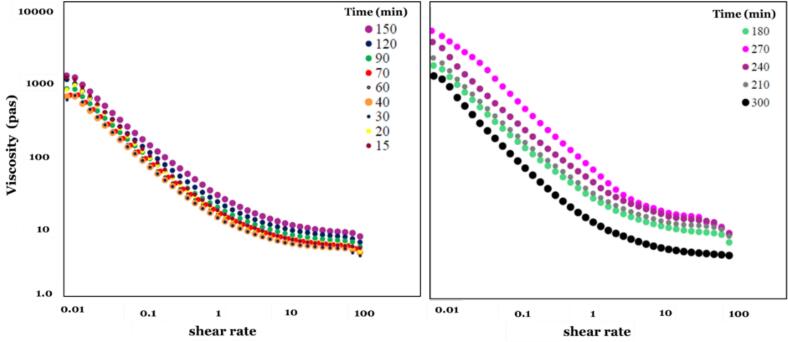
Apparent viscosity of the cream at different refining time points.

The rheological properties of the palm-sunflower-based anhydrous cream were markedly affected by refining duration. Across all treatments, samples exhibited characteristic pseudoplastic (shear-thinning) behavior, as evidenced by a progressive decline in apparent viscosity with increasing shear rate (Fig. 3). This response, which was accurately fitted by the Ostwald-de Waele model (*R*^*2*^ ≥ 0.99; Table 3), reflects the progressive alignment of dispersed solids and lipid domains under shear, thereby reducing interparticle friction and promoting flow under high deformation (Chaves et al., 2024; Rahmati et al., 2023). Such behavior represents a hallmark of concentrated fat-based dispersions, where the rheological profile is governed by both particle distribution and the viscosity of the continuous lipid phase.

**Table 3:** Flow rate and index of consistency of the cream according to the refining time.

Refining time (min)	n	K (Pa·s^n^)	*R*^*2*^
15	0.77 ± 0.00	13.59 ± 0.11	0.994
20	0.79 ± 0.00	13.61 ± 1.73	0.995
30	0.81 ± 0.00	11.25 ± 0.02	0.995
40	0.81 ± 0.00	11.48 ± 0.16	0.994
60	0.80 ± 0.00	12.95 ± 0.11	0.995
70	0.80 ± 0.00	13.02 ± 0.11	0.995
90	0.79 ± 0.00	15.77 ± 0.12	0.994
120	0.77 ± 0.01	18.72 ± 0.42	0.995
150	0.77 ± 0.01	19.34 ± 1.68	0.994
180	0.76 ± 0.00	26.97 ± 0.99	0.836
210	0.77 ± 0.00	30.80 ± 1.44	0.992
240	0.73 ± 0.00	41.49 ± 2.33	0.991
270	0.66 ± 0.02	54.82 ± 1.90	0.973
300	0.64 ± 0.02	73.37 ± 5.71	0.968

Refining duration exerted a pronounced effect on the rheological parameters. The consistency index (K) increased from 13.59 ± 0.11 Pa·sⁿ at 15 min to 26.97 ± 0.99 Pa·sⁿ at 180 min, and further to 73.37 ± 5.71 Pa·sⁿ at 300 min. In parallel, the flow behavior index (n) declined from 0.77 to 0.64, indicating increased shear-thinning behavior. These changes were closely associated with particle size reduction, which expanded the effective surface area and strengthened interactions between dispersed solids and the lipid matrix. When particle size approached ∼28 μm (D90) after 180–240 min, further refining produced negligible additional size reduction, yet viscosity continued to rise, reflecting intensified particle-particle interactions and a reduction in free volume (Borriello et al., 2022; Martínez-Padilla, 2024).

The rheological evolution observed in this study is consistent with previous findings on fat-based creams and spreads. Moderate increases in K coupled with decreases in n have been shown to enhance texture and spreadability, whereas excessive thickening reduces consumer acceptability (Borriello et al., 2022; Chaves et al., 2024). In the present system, refining for 180–240 min yielded the most favorable balance between particle size, viscosity, and flow behavior. Within this interval, K remained below ∼50 Pa·sⁿ, n was maintained within the acceptable range (0.70–0.76), and particle size stabilized, collectively supporting desirable creaminess and ease of spreading. Beyond this threshold (270–300 min), viscosity values exceeded 50 Pa·sⁿ, resulting in higher energy demands during pumping and mixing, greater processing inefficiency, and no additional sensory benefits. Therefore, 180–240 min can be regarded as the technological optimum, as it enhances creaminess and spreadability while preserving industrial processability.

### 3.3 Oxidation indices

Oxidative stability is a critical determinant of shelf life and overall quality in fat-rich cream systems, as lipid degradation typically results in rancidity, nutrient depletion, and the development of off-flavors (Zhuang et al., 2022). In this study, oxidative stability was evaluated at refining intervals of 0, 90, 150, and 300 min using free fatty acid (FFA) content and peroxide value (PV) as primary markers (Table 4). Results revealed no statistically significant differences across the refining durations, with FFA values remaining within a narrow range (0.75–0.84 %) and PV values between 25.4 and 26.7 meq O₂/kg oil. These findings indicate that refining at a controlled temperature of 45 °C did not induce oxidative degradation in the cream matrix, even under prolonged mechanical shear.

**Table 4:** Acidity and peroxide results of cream at different refining time.

	Replicate	Refining time (min)			
		0	90	150	300
**Acidity%**	1	0.81	0.85	0.79	0.74
	2	0.79	0.82	0.81	0.77
	**Average**	**0.80**	**0.84**	**0.80**	**0.75**
**Peroxide** **(meqO**_**2**_**/kg**_**oi**_**)**	1	26.28	25.72	25.94	26.45
	2	25.92	25.96	24.94	26.93
	**Average**	**26.10**	**25.84**	**25.44**	**26.69**

The observed oxidative stability can be attributed to formulation-specific factors, particularly the use of palm oil as the primary lipid source. Palm oil contains a higher proportion of saturated fatty acids and fewer polyunsaturated fractions, which enhances its oxidative stability. In contrast, oils such as linseed or sesame are highly prone to degradation, and their instability in cream systems has been highlighted in recent studies (Golmakani, Soltani and Sahraeian, 2024; Shuai et al., 2024). In addition, the closed-system and thermally controlled refining process minimized oxygen ingress and temperature fluctuations, both of which are well recognized as key accelerators of lipid oxidation (Geng et al., 2023). Taken together, the present data confirm that the anhydrous cream formulation preserved lipid stability across refining durations, supporting its potential for clean-label applications with extended shelf life. Combined with favorable rheological and granulometric properties, these findings underscore the suitability of palm–sunflower oil blends in the development of trans-fat-free cream systems for confectionery and frozen dessert applications.

### 3.4 Thermal properties of cream in ice cream application

Thermal behavior is a key quality determinant in fat-based systems for frozen and temperature-sensitive applications, as it governs melting profile, textural stability, and overall product performance. Differential scanning calorimetry (DSC) was used to evaluate the impact of refining duration on melting behavior. Samples refined for 0 and 300 min exhibited comparable thermograms, with onset temperatures around ˗56.0 °C and peak melting points at ˗27.4 °C and − 30.4 °C, respectively. The melting range extended to 6.4 °C in the 300 min sample, while enthalpy values remained stable at ∼23.8 J/g fat. These findings indicate that refining duration had negligible influence on thermal transitions, suggesting that the fat matrix retained structural stability despite prolonged shear.

**Fig. 4:**
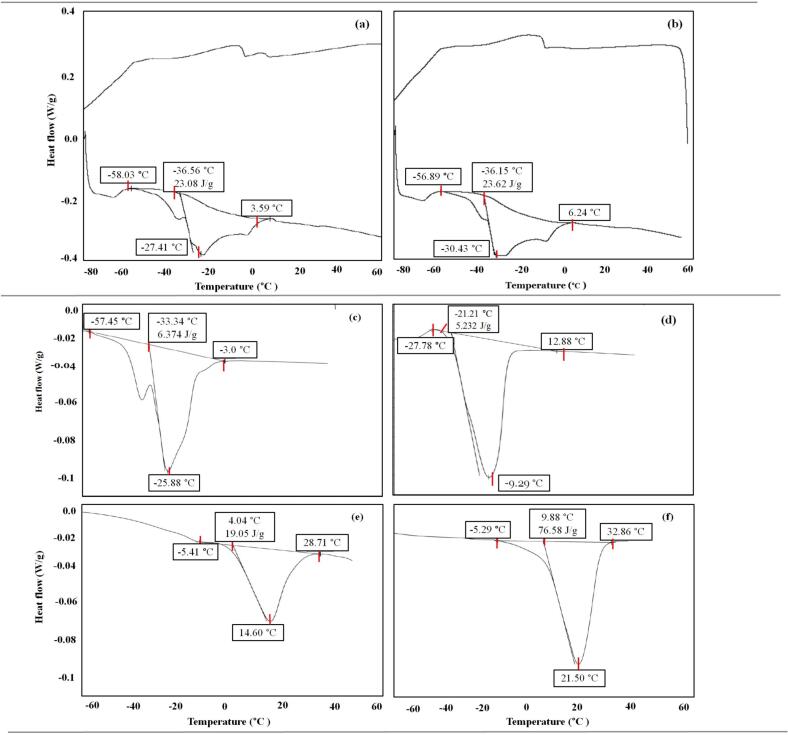
Thermogram of cream after (a) 0 min of refining time's (b) after 300 min of refining time, (c) sunflower oil thermogram, (d) Hazelnut cream thermogram, (e) palm oil thermogram and (f) cocoa butter thermogram. Red lines indicate the important thermal events. (For interpretation of the references to colour in this figure legend, the reader is referred to the web version of this article.)

The thermal profile reflects the triacylglycerol composition of the palm-sunflower blend, where both high- and low-melting fractions contribute to stable melting transitions without undesirable polymorphic shifts (Islam et al., 2022). The observed curves closely resembled those of cocoa butter and palm oil (Fig. 4e and f), with no evidence of recrystallization, secondary peaks, or phase separations. Such consistency is advantageous for frozen dessert applications, where structural integrity at storage and serving temperatures is critical.

From a product development perspective, thermal stability is highly desirable in applications such as ice cream inclusions, spreads, and confectionery fillings, where temperature fluctuations are common. Recent research has shown that palm-sunflower and related vegetable oil blends can replicate the thermal and solid fat content behavior of milk fat, thereby ensuring functionality in frozen dessert systems (Hasan et al., 2022). Overall, the cream formulation exhibited a stable melting profile without signs of degradation or fat bloom, reinforcing its suitability for clean-label, trans-fat-free formulations in frozen and ambient-stable food products.

## 4 Conclusion

This study demonstrates that refining duration in a stirred ball mill is a decisive factor in tailoring the physicochemical and functional properties of palm-sunflower anhydrous cream. Specifically, particle size reduction stabilized at D90 ≈ 28–30 μm after 180–240 min; however, further milling beyond this threshold provided negligible improvement. Within this interval, the consistency index (K) increased to ∼27 Pa·sⁿ while the flow behavior index (n) declined to ∼0.76, thereby confirming a desirable shear-thinning profile that enhances creaminess and spreadability. In contrast, prolonged refining to 270–300 min caused excessive thickening (K > 50 Pa·sⁿ; *n* < 0.65), which increased energy demand and complicated industrial handling without additional sensory benefits. Moreover, oxidative stability (PV 25–27 meq O₂/kg; FFA 0.75–0.84 %) and thermal transitions remained stable, indicating that extended refining did not compromise lipid integrity.

Consequently, the findings identify 180–240 min as the optimal processing window, offering a balance between particle fineness, rheological quality, oxidative stability, and energy efficiency. Overall, this benchmark provides practical guidance for manufacturers seeking clean-label, trans-fat-free fat systems suitable for confectionery, spreads, chocolate fillings, and frozen desserts. Beyond its technological relevance, the study expands current understanding of stirred ball mill refining by applying it to palm-sunflower cream, highlighting its significance as a promising clean-label fat system. Future research should therefore prioritize sensory validation, pilot-scale trials, and storage stability assessments to directly link rheological thresholds with consumer acceptability and long-term performance under industrial conditions.

## CRediT authorship contribution statement

**Mohammad Salama:** Writing – original draft, Methodology, Investigation, Formal analysis, Conceptualization. **Birsen Sarıcı:** Writing – review & editing, Supervision, Methodology, Conceptualization. **Avni Çakıcı:** Writing – review & editing, Supervision, Methodology, Investigation, Formal analysis.

## Ethics statement

This study did not involve human participants or animal experiments.

## Funding

This research received no specific grant from any funding agency in the public, commercial, or not-for-profit sectors.

## Declaration of competing interest

The authors declare that they have no known competing financial interests or personal relationships that could have appeared to influence the work reported in this paper.

## Data Availability

The data that support the findings of this study are available from the corresponding author upon request.
